# Optical and Flame-Retardant Properties of a Series of Polyimides Containing Side Chained Bulky Phosphaphenanthrene Units

**DOI:** 10.3390/ijms232113174

**Published:** 2022-10-29

**Authors:** Mihaela Homocianu, Diana Serbezeanu, Gabriela Lisa, Mihai Brebu, Tăchiță Vlad-Bubulac

**Affiliations:** 1 “Petru Poni” Institute of Macromolecular Chemistry, Aleea Gr. Ghica Voda, 41A, 700487 Iasi, Romania; 2 Department of Chemical Engineering, Faculty of Chemical Engineering and Environmental Protection, Gheorghe Asachi Technical University of Iasi, Bd. Mangeron 73, 700050 Iasi, Romania

**Keywords:** polyimides, DOPO (9,10-dihydro-9-oxa-10-phosphaphenanthrene-10-oxide), thermal decomposition mechanism, Py-GC, char residue, SEM-EDX

## Abstract

Among the multitude of polymers with carbon-based macromolecular architectures that easily ignite in certain applications where short circuits may occur, polyimide has evolved as a class of polymers with high thermal stability while exhibiting intrinsic flame retardancy at elevated temperatures via a char-forming mechanism. However, high amounts of aromatic rings in the macromolecular backbone are required for these results, which may affect other properties such as film-forming capacity or mechanical properties; thus, much work has been done to structurally derivatize or make hybrid polyimide systems. In this respect, flexible polyimide films (PI(1–4)) containing bulky 9,10-dihydro-9-oxa-10-phosphaphenanthrene-10-oxide (DOPO) units have been developed starting from commercial dianhydrides and an aromatic diamine containing two side chain bulky DOPO groups. The chemical structure of PI(1–4)) was characterized by ^1^H NMR, ^13^C NMR and ^31^P NMR spectroscopy. The optical properties, including absorption and luminescence spectra of these polymers, were analyzed. All polyimides containing DOPO derivatives emitted blue light with an emission maxima in the range of 340–445 nm, in solvents such as N,N-dimethylformamide, N-methyl-2-pyrrolidone, chloroform, and N,N-dimethylacetamide, while green light emission (**λ**_em_ = 487 nm for PI-4) was evidenced in a thin-film state. The thermal decomposition mechanism and flame-retardant behavior of the resulting materials were investigated by pyrolysis-gas-chromatography spectrometry (Py-GC), scanning electron microscopy (SEM), EDX maps and FTIR spectroscopy. The residues resulting from the TGA experiments were examined by SEM microscopy images and FTIR spectra to understand the pyrolysis mechanism.

## 1. Introduction

Polyimides, polymers with multiple commercially available representatives, continue to be a class of high-performance polymers of great interest to the scientific community [1,2,3]. These polymers are being studied more and more because of their desirable features, including outstanding thermo-oxidative stability, intrinsic flame resistance, distinct electrical characteristics, excellent radiation and solvent resistance, and good mechanical qualities [4,5]. Despite their unique characteristics the extensive use of fully aromatic polyimides is limited mainly due to their advanced insolubility in organic solvents [6,7]. The increasing need to improve the processability of polyimides has prompted researchers to create new structures with various flexible linkages, bulky substituents, alicyclic units, or non-coplanar structures, thus, a range of features has been designed to fulfill certain industry requirements [8,9]. DOPO and its derivatives with voluminous phosphaphenanthrene groups in their structures have appeared as a solution to the development of polymers with increased solubility and thermal stability due to the presence of the P–O–C bond [10,11,12]. Furthermore, interesting optical properties have been reported for such DOPO-containing high performance polymers [13,14,15,16]. To our knowledge, DOPO-containing polyimides have rarely been developed into flexible thin films as a consequence of the bulky nature of DOPO, which may prevent molecular packing [17,18].

Among the materials with excellent flame-retardant properties, the organophosphorus compounds have been much studied recently [19,20]. Xie et al. [21] prepared 9,10-dihydro-9-oxa-10-phosphaphenanthrene-10-oxide derivative grafted polyethylene films to improve the flame retardant and anti-dripping properties. They found that, compared with pure PE film, the burning rate of anti-dripping DOPO-t/DOPO-m functionalized PE films was reduced by 13.2% and 50.0%, and the limiting oxygen index value climbed to 18.5 and 19.5, respectively. The residual chars from DOPO-t/DOPO-m functionalized PE films at 700 °C were increased. Polyurethane containing DOPO and siloxane [22] derivatives have also shown remarkable flame-retardant and smoke-suppressant performances. The synergistic effect of phosphorus and silicon in these derivatives induce enhanced flame retardancy and also increased the limiting oxygen index from 17.0% to 26.1% but also reduced the total smoke release by 17.5%.

Aromatic polyimide derivatives containing –CH_3_ or –CF_3_ groups have effectively higher solubility, free volume, inherent fire-resistant properties, increased thermal stability, and high polarizability, which are essential for industrial applications. Recently, He et al. [16] synthesized an imide-DOPO derivative with excellent flame retardancy and aggregation-induced emission enhancement, simultaneously exhibiting fluorescence properties without losing their glass transition temperature. In another study, Jiang et al. [23] studied the effects of cardanol-based co-curing agents with different phosphorus structures on the mechanical and flame-retardant properties of bismaleimide resin. The thermal stability, flame retardancy and mechanical performance of the produced bismaleimide were greatly modified by the presence of the DOPO-cardanol modifiers. The authors found that the phosphorylated cardanol with higher content of O and higher valence of P can be used to improve the mechanical property and fire safety of bismaleimide [24]. Polyimides and polyamides containing DOPO with excellent solubility and attractive thermal characteristics have also been reported by Y.-L. Liu at al. [25,26]. Their studies noticed that the addition of the DOPO group to polyimides or polyamides improved their weight loss rate, thermal-oxidative stability, and heat insulating property at high temperatures, but more research is needed to determine film forming ability, optical properties, pyrolysis mechanisms and reaction-to-fire behavior.

In our previous studies, thermal and flame-retardant properties of a series of phosphorus-containing polyimide powders were introspected by means of thermogravimetric analysis (TGA) and pyrolysis combustion flow calorimetry (PCFC), while Fourier transform infrared spectroscopy (FTIR) and scanning electron microscopy (SEM) were applied to establish that their remarkable char yields at 900 °C, ranging from 35% to 54%, play an important role in the degradation mechanism of these polyimides [27].

**Figure 1:**
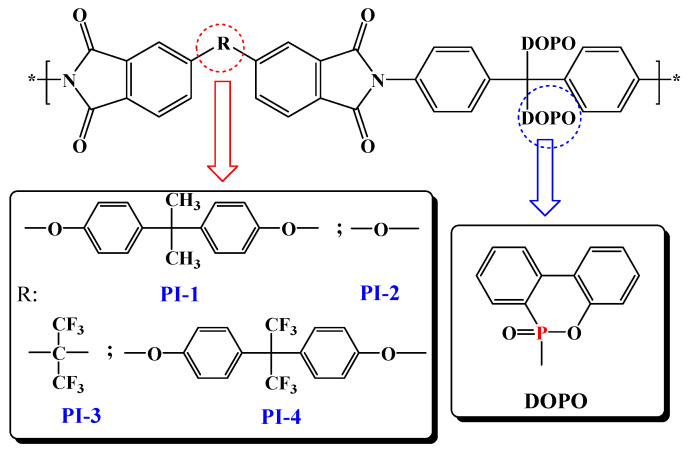
Schematic representation of the PI(1–4) chemical structures.

On continuing our efforts to enlighten high performance characteristics, such as photophysical and photoluminescence properties, and to further understand the mechanism of thermal decomposition of this type of polyimide that contains two side chained bulky phosphaphenanthrene groups per structural unit, a series of flexible films based on DOPO-containing polyimides, PI(1–4) were developed in the current study (Figure 1). Optical properties were introspected by recording absorption and photoluminescence spectra, both in various solutions or in film state, while the thermal decomposition mechanism and the flame-retardant behavior of the PI(1–4) films were analyzed by means of pyrolysis-gas chromatography-mass spectroscopy (Py-GC-MS), scanning electron microscopy, and FTIR spectroscopy data of the char yield at different temperatures.

## 2. Results and Discussion

### 2.1. Optical Properties of DOPO-Containing Polyimides

**Figure 2:**
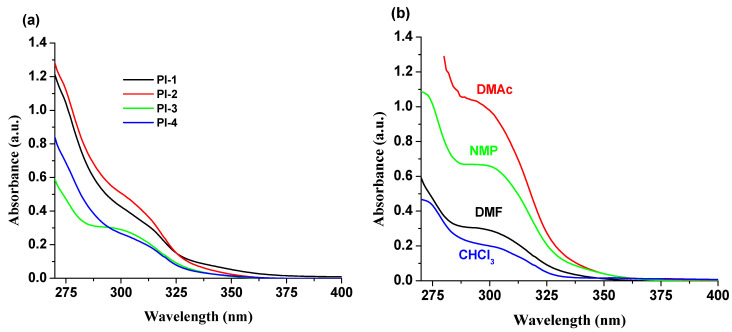
Absorption spectra of (**a**) PI(1–4) derivatives in DMF and (**b**) PI-3 in NMP, DMF, CHCl_3_ and DMAc solvents.

Absorption spectra of PI(1–4) polyimides in DMF, NMP, CHCl_3_ and DMAc solvents were recorded and studied. The summary of the obtained photophysical characteristics is shown in Table 1. In all solvents, an absorption band in the range of 275–350 nm was observed (Table 1 and Figure S1, Supporting Information), which is strongly influenced by DOPO units [28], but the other different structural groups (-O-C_6_H_5_-C(CH_3_)_2_-C_6_H_5_-O- (for PI-1), -O- (for PI-2), -C(CH_3_)_2_- (for PI-3), and -O-C_6_H_5_-C(CF_3_)_2_-C_6_H_5_-O- (for PI-4)) in these compounds have a slight effect on the absorption spectra (Figure 2a). The PI-3 sample, containing the electron withdrawing -C(CF_3_)_2_- unit into the structure, shows a well-structured absorption band around 300 nm (Figure 2b), compared to the other DOPO-containing polyimides under study. The solvent effects on the band position are minor, i.e., a 4–6 nm bathochromic shift with increasing polarity of the solvent.

### 2.2. Photoluminescence Properties

**Figure 3:**
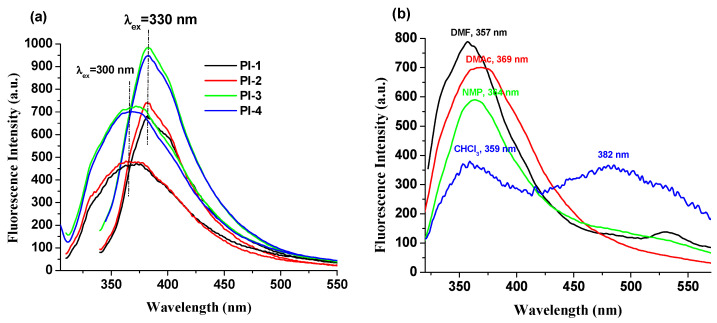
Emission spectra of (**a**) PI(1–4) in DMAc solutions and (**b**) PI-4 in NMP, DMF, CHCl_3_ and DMAc solutions (when the excitation wavelength was 300 nm).

The fluorescence properties of the PI(1–4) in DMF, NMP, CHCl3 and DMAc solvents and in solid state (thin films) were explored. These compounds exhibit an emission band (Table 1) that is influenced by the values of the excitation wavelengths. For example, PI-1 displayed emission at 373 nm (λ_ex_ = 300 nm) and 382 nm (λ_ex_ = 330 nm) in DMAc solution, respectively (Figure 3a). No significant and clear relationship is observed between the electronic effects of the substituents (-O-C_6_H_5_-C(CH_3_)_2_-C_6_H_5_-O- (for PI-1), -O- (for PI-2), -C(CH_3_)_2_- (for PI-3), and -O-C_6_H_5_-C(CF_3_)_2_-C_6_H_5_-O- (for PI-4)) and the luminescence band shifts (Figure 3b). This behavior indicates that these substituents seem to slightly influence the emission of PI(1–4) samples, which may be due to the long distance between the substituent groups and DOPO units.

The chloroform solutions of these DOPO–containing polyimides exhibit a distinctive fluorescence profile because the weak polarity of the chloroform blocks the formation of highly polar twisted intramolecular charge transfer TICT [29]. Moreover, in this solvent, two emission bands appear for all samples (Table 1, Figure 3 and Figure S3), whose position is independent of the excitation wavelength values.

### 2.3. Excitation-Dependent Emission Property

**Figure 4:**
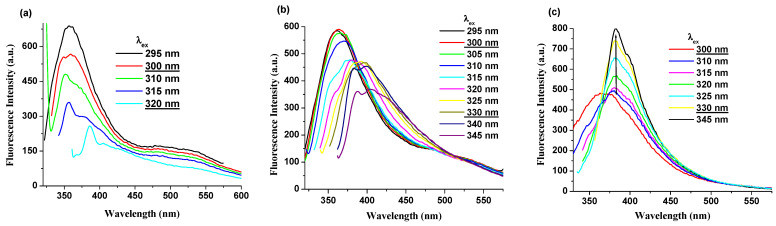
Photoluminescence spectra, excited at different wavelengths, of (**a**) PI-1 in DMF solution, (**b**) PI-4 in NMP solution, and (**c**) PI-2 in DMAc solution.

Photoluminescence data of PI(1–4) derivatives were analyzed under various excitation wavelengths and solvents. Detailed fluorescence spectra in selected solvents at different excitation wavelengths are shown in Figure 4 and Figures S2–S5, respectively. Under the same excitation wavelengths (300–330 nm), the PI(1–4) displays a decrease in the intensity of the photoluminescence band in DMF, NMP and CHCl_3_ solutions (Figure 4a and Figures S2–S4). Instead, in the DMAc (ε = 37.78) solution, along with an increase in excitation wavelength (from 300 to 330 nm) its fluorescence intensity increases significantly and the shape of the emission band becomes narrower, as shown in Figure 4c and Figure S5 from Supporting Information. Moreover, it was found that in all solutions the position of the λ_em_ was easily red shifted along with an increase in excitation wavelength (from 300 to 330 nm).

**Figure 5:**
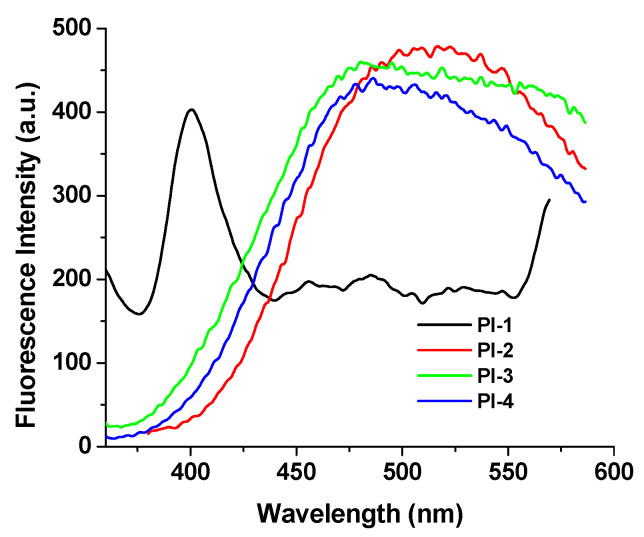
Solid-state emission spectra of PI(1–4) at room temperature (when the excitation wavelength is 330 nm).

As shown in Figure 5, the film state emits a strong red-shifted fluorescence (with λ_em_ at above 510 nm, except for PI-1) quite different from that in solutions (Table 1) where the polymer chains underwent an expansion.

The differences found in the λ_em_ position and the shape of the emission spectra of the PI-1 thin film can probably be due to the characteristics of the transmission spectra for this sample with higher optical transmittance than the other films, see Figure S6 in the Support Information.

### 2.4. Py-GC-MS Analysis

**Figure 6:**
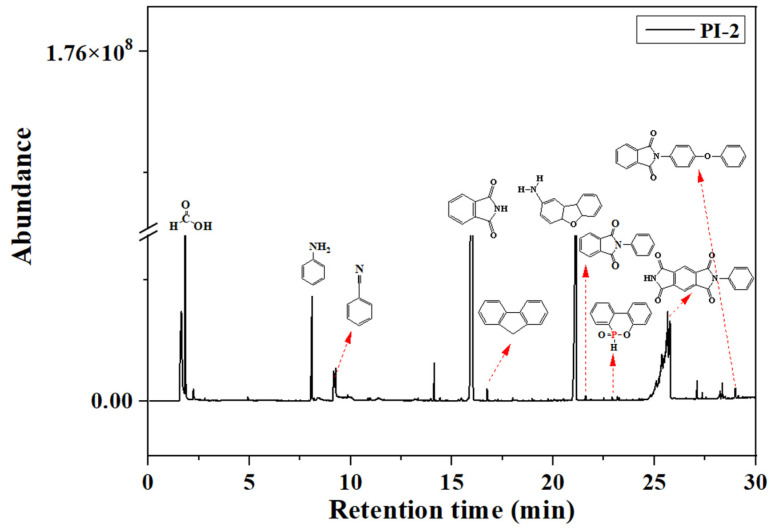
Py/GC chromatogram of the PI-2 at 600 °C.

Pyrolysis, followed by GC-MS analysis, was performed in order to determine the decomposition mechanisms of the PI(1–4) based on the products resulting from thermal scission of chemical bonds. Figure 6 exhibits the total ion chromatogram of the pyrolysis products of the PI-2 sample, as an example in the series. In accordance with the increasing retention times (RT, min.) it can be observed that the main pyrolysis products of PI-2, are (Figure 6): formic acid (1.814 min.), benzene (2.797 min.), toluene (4.216 min.), aniline (8.037 min.), benzonitrile (8.130 min.), naphthalene (11.350 min.), dibenzofuran (15.470 min.), phthalimide (16.300 min.), fluorine (16.840 min.), p-hydroxybiphenyl (18.140 min.), and phenanthrene (19.10 min.). Other pyrolysis products revealed in the chromatogram are 4a,5a,9a,9b-tetrahydrodibenzo[b,d]furan-2-amine (RT = 20.74), 2-phenylisoindoline-1,3-dione/1H-isoindole-1,3(2H)-dione 2-phenyl (RT = 21.59), 9,10-dihydro-9-oxa-10-phosphaphenanthrene-10-oxide (RT = 22.91), 2-phenylpyrrolo [3,4-f]isoindole-1,3,5,7(2H,6H)-tetraone (RT = 26.75), and 2-(4-phenoxyphenyl) isoindoline-1,3-dione (RT = 29.00) [30,31,32,33].

At a higher temperature (600 °C), although phosphaphenanthrene heterocycle could still be detected as a product, the pyrolysis of DOPO rings further degrades to form dibenzo[b,d]furane and 1,1’-biphenyl; two compounds which were released by cleavage of P–C bonds (from DOPO). This behavior has also been noticed by other authors [34,35] and was observed for all the samples in the current study.

Furthermore, it is well known that during the pyrolysis process, the organophosphonic flame retardants release PO·, HPO· and HPO_2_· in the gas phase. As a consequence, recombination mechanisms may occur. Thus, according to data obtained from TIC, DOPO rings could rearrange in fluorene (RT = 16.844/16.764/16.740), biphenyl (RT = 14.141/14.124), dibenzofuran (RT = 27.115/25.651/25.655) and o-hydroxybiphenyl (RT = 16.10/15.972/16.02). Also, in all three samples the presence of the NMP (RT = 11.175/10.082/9.255) from TIC may be attributed to the evaporation of the residual solvent encapsulated in the PI’s macromolecular chain. The presence of the 3-(trifluoromethyl)phenol was observed in the TIC of sample PI-3 at RT = 15.048 [36].

**Figure 7:**
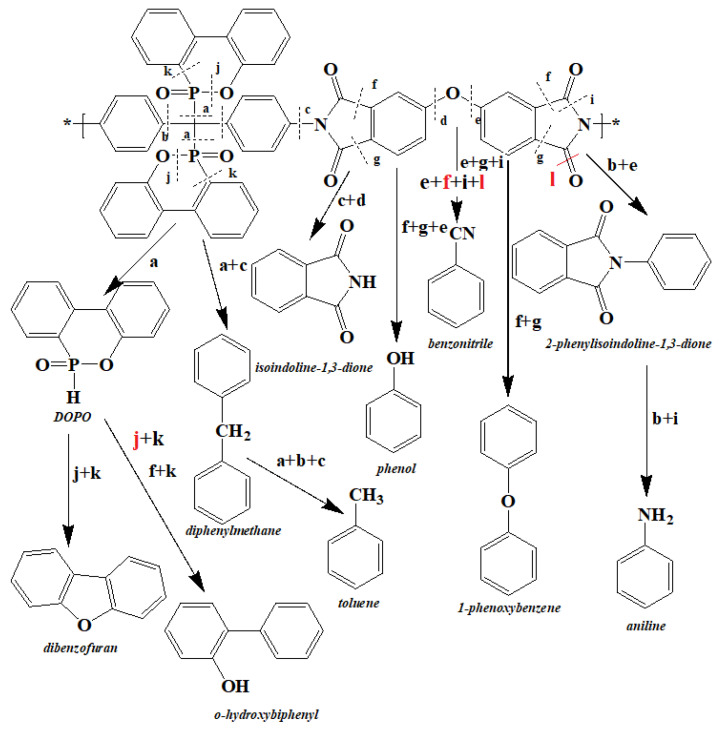
The proposed degradation mechanism for the PI-2 polymer.

Based on the results obtained from mass loss, the analysis of the released gasses and the products remaining in the condensed phase, the decomposition mechanism of the phosphorus-containing polyimides (PI-2) was proposed (Figure 7).

### 2.5. Char Residue Investigation

#### 2.5.1. Morphological Analysis of the Char Residue

**Figure 8:**
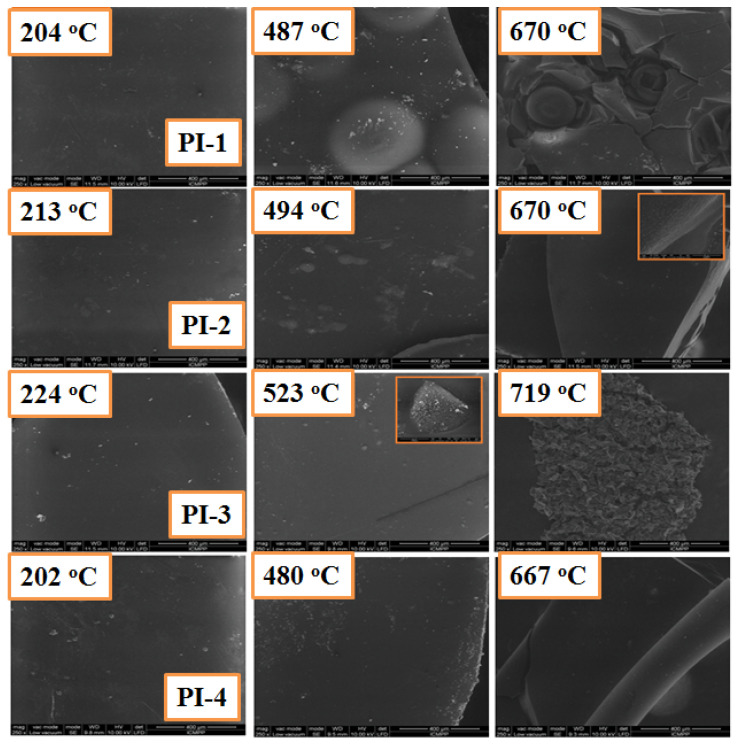
SEM images for carbonaceous residues of PI(1–4) at different degradation stages under a nitrogen atmosphere, which were obtained from TGA experiments.

The SEM images of the carbonaceous residues for PI(1–4) polymers obtained after heating the samples at different temperatures are shown in Figure 8. It can be seen that the surface of the char residue exhibits a dense structure without cracks or pores at the end of the first degradation stage. When the degradation is finished, the char residue still shows a continuous and dense structure, but the residue obtained for PI-2 and PI-4 has a much more compact structure compared to the PI-1 and PI-3 polymer residues, respectively, which indicates that the aliphatic sequences of PI-1 polymers are eliminated during the first stage of thermal decomposition, penetrating through the resulting carbonaceous residue and leading to a solid coal with a less compact structure. This behavior was also seen for the PI-3 polyimide.

**Figure 9:**
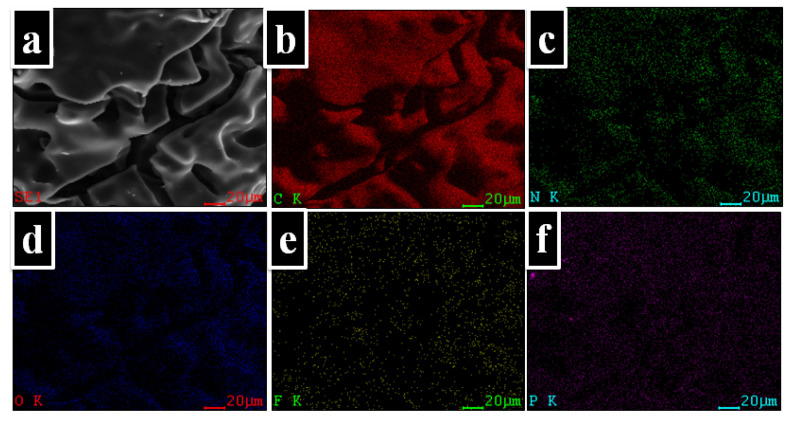
EDX mappings of the char yields of PI-3 at 900 °C. (**a**) SEM images for carbonaceous residues of 900 °C; (**b**) distribution of carbon atoms (C: carbon); (**c**) distribution of nitrogen atoms (N: nitrogen); (**d**) distribution of oxygen atoms (O: oxygen); (**e**) distribution of fluor atoms (F: fluor); (**f**) distribution of phosphorus atoms (P: phosphorus).

A mapping technique was used to observe the distribution of the atoms onto the char residue surface. Thus, in Figure 9 can be observed the elemental composition of the PI-3 as it was identified by EDX analysis. Furthermore, as shown in the elemental mapping images, these elements are uniformly dispersed in the char residue. Also, from Figure 8, it can be observed that the P is homogeneously dispersed on the surface of the char residue.

**Figure 10:**
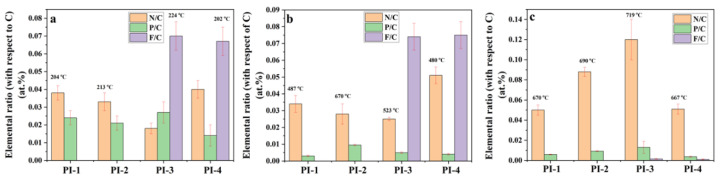
Elemental N/C, P/C and F/C ratios (according to EDX analysis) of PI(1–4) at different degradation stages under nitrogen atmosphere obtained from TGA experiments (**a**) at 204(PI-1)/213 (PI-2)/224 (PI-3)/202 (PI-4) °C, (**b**) at 487 (PI-1)/670 (PI-2)/523 (PI-3)/480 (PI-4) °C and (**c**) at 670 (PI-1)/690 (PI-2)/719 (PI-3)/667 (PI-4) °C.

The atomic distribution and concentration of N, P, F, and C of char residues was also analyzed using the SEM-EDX microanalysis. From Figure 10 it can be observed that the P/C and F/C ratios decreased with increasing temperature, while N/C ratio increased with temperature, data which are consistent with the results obtained by Py-GC-MS. These data can be explained by the decomposition of PI(1–4) and the removal of phosphorus oxides in the form of a gaseous product at high temperatures. The EDX analysis of PI(1–4) above confirmed that the P/C ratio decreased after the second stage of decomposition (Figure 10). Thus, the reasons for the appearance of large pores could be attributed to the evaporation of the trifluoromethane (CHF_3_) which leads to the pore formation in the solid phase. The evaporation of CHF_3_, which starts in the second step of decomposition, leads to a “spongy” material. If the temperature does not exceed 523 °C, then the first process prevails, while temperature increases above this value give evidence of pores.

#### 2.5.2. FTIR Analysis of the Char Residue

**Figure 11:**
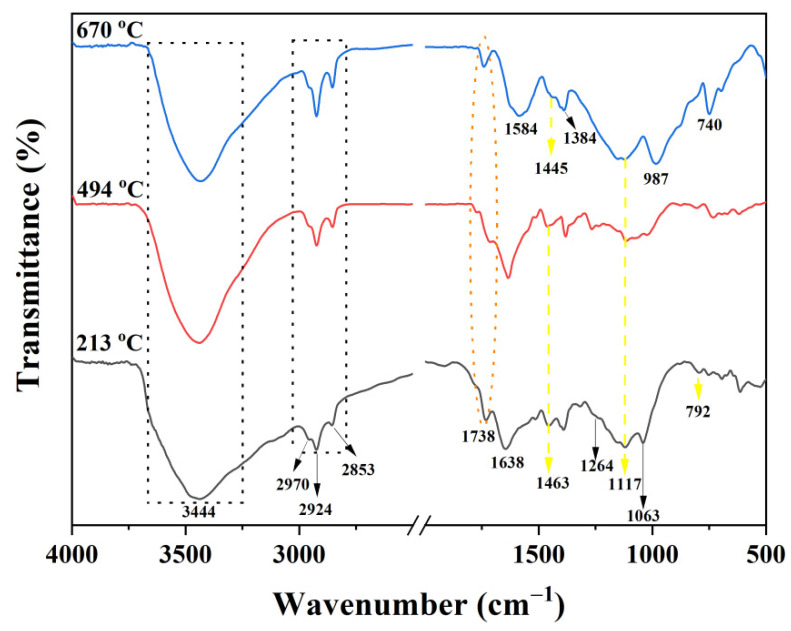
FTIR spectra of the char residue of PI-2 at different degradation stages (213 °C, 494 °C and 670 °C) under nitrogen atmosphere TGA experiment.

FTIR spectra at different temperatures for the char residues of the PI-2 resulting from the TGA experiments are exhibited in Figure 11. The characteristic absorption peaks of PI-2 at 213 and 494 °C are at 1738 cm^−1^ (C=O stretching vibrations), 3444 cm^−1^ (-OH stretching vibrations), 2970, 2924 and 2853 cm^−1^ (C-H stretching vibrations), 1264 and 1063 cm^−1^ (C-O-C), and 1463, 1117 and 792 cm^−1^ (P-Ar, P=O and P-O-Ar). The presence of the phosphorus in the char residue confirms the results obtained from the Py-GC-MS and SEM; therefore, the P-containing char residue is formed in the later stage of decomposition (first stage of decomposition from TGA experiment). In the FTIR spectrum of PI-2 at 494 °C weak absorption bands between 1600 and 1300 cm^−1^ can be observed, this weak absorption band at a temperature of 670 °C becomes stronger and could be attributed to the molecular chain scission [37]. At 670 °C, the degradation of the PI-2 occurs and in the FTIR spectrum, it can be observed only at the following absorption bands: 3430 cm^−1^ (-OH), 1584 cm^−1^ (C=C), 1445 cm^−1^ (P-Ar), 1384 cm^−1^ (C-H), 1117 cm^−1^ (P=O), 987 cm^−1^ (P-O-Ar) and 740 cm^−1^ (imide ring deformation). According to these data, the phosphorus is still present in the solid residue. The absorption band at 1584 cm^−1^ indicates the presence of the aromatic compounds in the char residue, results that are in agreement with the Py-GC-MS and SEM data. Further, the new bands appeared subsequently at 1584 cm^−1^ and 1384 cm^−1^, indicating that the main chains of PI-2 were broken, thus also leading to the formation of graphite char residue data which is in agreement with the SEM images (Figure 8). The absorption peaks at 1117 cm^−1^ and 740 cm^−1^ belong to PO_2_ or PO_3_ in phosphate carbon complexes, while the peak at 987 cm^−1^ was assigned to the P-O-Ar, indicating char residues, such as P_2_O_5_ and P_4_O_10_.

## 3. Materials and Methods

### 3.1. Materials

4,4’-(4,4’-Isopropylidenediphenoxy)bis(phthalic anhydride) (98%, Mw = 41,051 g/mol), 4,4′-oxydiphthalic anhydride (97%, Mw = 31,021 g/mol), 4,4’-(hexafluoroisopropylidene) diphthalic anhydride (99%, Mw = 44,424 g/mol), 4,4’-[(hexafluoroisopropylidene)bis[(4,1-phenylene)oxy]]bis(isobenzofuran-1,3-dione) (97%, Mw = 628.4 g/mol), 4,4’-diaminobenzophenone (97%, Mw = 21,225 g/mol), and N-methyl pyrrolidone (NMP) (HPLC, 99.9% purity) from Sigma-Aldrich Chemie GmbH were used as received. 9,10-dihydro-9-oxa-10-phosphaphenanthrene-10-oxide (DOPO) (97%, Mw = 216.18 g/mol) received from TCI (Japan) was dehydrated under vacuum for 5 h at 120 °C prior to use. The other solvents used in the synthesis, to precipitate or purify the polymers, were of analytical grade and used as received.

2DOPO-NH_2_ was synthesized according to previously published procedures starting from 4,4’-diaminobenzophenone and 9,10-dihydro-9-oxa-10-phosphaphenanthrene- 10-oxide [24,25,27].

Yield: 75%.
^1^H NMR (DMSO-*d6*, ppm): δ = 4.9 (s, 4H, NH_2_), 5.9 (d, 4H, CHar-NH_2_), 8.5–6.9 (m, 20H, aromatic protons);
^31^P NMR (DMSO-*d6*, ppm): δ = 31.10 and 29.41.

### 3.2. Synthesis of Phosphorus-Containing Polyimides and Preparation of DOPO-Containing Polyimide Films

The preparation of the PI(1–4) polyimides studied in the current work has been described in detail in our previously published paper [27]. Nevertheless, in the present work, the PI(1–4) have been prepared via casting from solutions technique followed by the thermal imidization in film state instead of thermal imidization in solution. A typical example is presented for the polyimide denoted as PI-4, which was synthesized by the polycondensation reaction in two steps of diamine denoted as 2DOPO-NH_2_, with commercial dianhydrides, namely, 4,4’-[(hexafluoroisopropylidene)bis[(4,1-phenylene)oxy]] bis(isobenzofuran-1,3-dione), using NMP as solvent (Figure 1). Typical procedure involves the introduction of 0.626 g (1 mmol) of 2DOPO-NH_2_ and 9.2 mL NMP in a three-necked 100 mL round flask, equipped with a magnetic stirrer and nitrogen inlet and outlet. After complete dissolution of the diamine, the mixture was cooled in an ice and salt bath, and then 0.628 g (1 mmol) of 4,4’-[(hexafluoroisopropylidene)bis[(4,1-phenylene)oxy]]bis(isobenzofuran-1,3-dione) were introduced under vigorous stirring. The reaction mixture was kept below 0 °C for 15 min, after which the reaction was carried out at room temperature for an additional 8 h. All the PI(1–4) polymers studied showed film forming ability. In order to obtain thin films, solutions of these polymers in NMP with concentrations of 12% (wt.%) were poured onto glass plates and dried for one hour at each of the following temperatures: 100 °C, 120 °C, 150 °C and 180 °C. By immersion in hot water the PI(1–4) films were stripped from the glass plate. After that, the films were maintained for 5 h in an oven at 200 °C. All PI(1–4) poyimide films were purified by extraction with ethanol in a Soxhlet. After purification and drying in an oven at 90 °C, films were obtained independently, with thicknesses in the range of 20–40 µm.

PI-1: ^31^P NMR (DMSO-*d6*, ppm): 29.90, 27.82;

PI-2: ^31^P NMR (DMSO-*d6*, ppm): 29.86, 27.76;

PI-3: ^31^P NMR (DMSO-*d6,* ppm): 29.91, 28.51;

PI-4: ^31^P NMR (DMSO-*d6,* ppm): 29.87, 28.32.

Basic characterization data regarding thermal properties and combustion behavior of the polyimides under study, including limiting oxygen index (LOI, 39.1%), carbonaceous residue yield measured at 600 °C (60–70%), carbonaceous residue yield measured at 900 °C (35–54%), heat release capacity (HRC, 109.37–165.28 J/g_*_K), and total heat release THR (8.14–11.30, kJ/g), were performed in our previous work [27].

### 3.3. Methods

The absorption spectra were obtained using an Analytic Jena 210+ spectrophotometer. The photoluminescence spectra in N,N-dimethylformamide, (DMF), N-methyl-2-pyrrolidone, (NMP), chloroform (CHCl_3_) and dimethylacetamide, (DMAc), both as solvents and in the solid state (thin films) were measured with a Perkin Elmer LS55 fluorescence spectrophotometer and by exciting the PI(1–4) polyimide samples with variable wavelengths from 295 nm to 345 nm. All spectra were performed at room temperature using quartz cuvettes with an optical path length of 10 mm.

FTIR spectral analysis of monomers 2DOPO-NH_2_, polymers PI(1–4) and char yields of polymers PI(1–4) obtained from the TGA analysis were performed with a Bruker Vertex 70 FTIR spectrometer, in transmission mode, using KBr tablets on frequency range 4000–400 cm^−1^ or in reflection mode, using polymeric films PI(1–4) with thickness between 20–40 µm in the range 4000–600 cm^−1^.

^1^H NMR, ^13^C NMR and ^31^P NMR measurements for monomers and polymers PI(1–4) were performed at room temperature using a spectrometer Bruker Avance DRX400, at various operating frequencies (400 MHz for ^1^H NMR, 100 MHz for ^13^C NMR and 62MHz for ^31^P NMR), in DMSO-*d_6_*.

The char yields were obtained by using Mettler Toledo TGA-SDTA851 equipment, in a nitrogen atmosphere, under dynamic conditions with a flow rate of 20 mL/min and a heating rate of 10 °C/min, using different temperature ranges and with a sample mass between 2 and 3 mg.

Scanning electron microscopy (SEM) of the char residue was performed on a Verios G4 UC Scanning Electron Microscope (Thermo Scientific, Czech Republic). The char yields were coated with 6 nm platinum using a Leica EM ACE200 Sputter coater to provide electrical conductivity and to prevent charge buildup during exposure to the electron beam. SEM investigations were performed in high vacuum mode using a secondary electron detector (Everhart-Thornley detector, ETD) at an accelerating voltage of 5 kV. In order to evaluate the elemental analysis of the char residue of PI(1–4) a coupled dispersive X-ray spectroscopy (EDX) was used.

About 50 mg samples were pyrolyzed at 550 °C in a tubular glass reactor with an internal diameter of 5 mm. A nitrogen flow removed the gaseous products of thermal degradation from the heated zone and transported them into a diethylether trap in which they were solubilized prior to characterization by gas chromatography coupled with mass spectrometry (GC-MSD). The GC-MSD was performed on a 6890N Agilent gas chromatograph coupled to a 5975 Inert XL Agilent mass detector (Agilent, Santa Clara, CA, USA), under the following conditions: HP5–MS capillary column (30 m × 0.25 mm × 0.25 μm), temperature program of 40 °C (2 min), 10 °C min^−1^ → 320 °C, helium flow of 1 mL min^−1^, inlet temperature of 250 °C, injected volume of 0.2 μL, split ratio of 100:1, and with a quality of recognition according to NIST 14 MS database of a minimum of 85%.

## 4. Conclusions

A series of DOPO-containing polyimides were successfully synthesized and characterized regarding their optical and reaction-to-fire properties. The optical properties of these compounds were investigated and discussed by absorption/photoluminescence spectra in different solvents (DMF, NMP, CHCl_3_ and DMAc) and solid state (films). The flame-retardant mechanism analysis was investigated by SEM-EDX, TG-FTIR and Py-GC-MS coupling technique, and the resulting data indicate that the high thermal stability led to several breakages of chemical bonds and, also, a decrease of surface quality was observed. Finally, new chemical bonds of C–C, CH_2_–O and CN were formed. A pyrolysis mechanism of the PI-2 was proposed and discussed. The FTIR results indicate that at 670 °C, degradation of the PI-2 occurs. FTIR and Py-GC-MS results demonstrate that PI-2 decomposed first in the early stage of degradation, generating pyrolysis products and forming phosphorus-containing char residue in the later stages of degradation.

## Figures and Tables

**Table 1:** Summary of photophysical spectral data.

Data		CHCl_3_	DMF	NMP	DMAc	Films
$a^{\;}\lambda_{\max}^{abs}$, nm	PI-1	274 ^sh^, 312	306	273 ^sh^, 311	310 ^sh^	-
	PI-2	274 ^sh^, 309	306	273 ^sh^, 311	307 ^sh^	-
	PI-3	274 ^sh^, 304	302	273 ^sh^, 303	311	-
	PI-4	274 ^sh^, 309	311	273 ^sh^, 310	300 ^sh^	-
$b^{\;}\lambda_{\max}^{em}$, nm	PI-1	362, 481	* 357, ** 368	* 365, ** 395	* 373, ** 384	400
	PI-2	357, 493	* 359, ** 368	* 364, ** 394	* 363, ** 384	515
	PI-3	354, 470	* 356, ** 369	* 372, ** 382	* 370, ** 383	481
	PI-4	359, 382	* 357, ** 369	* 364, ** 395	* 369, ** 383	487

**^a^**$\lambda_{\max}^{abs}$ (nm)—the wavelength of the absorption maximum; **^b^**$\lambda_{\max}^{em}$ (nm)—wavelength of the emission maximum (monitoring wavelength of emission for excitation spectra); *—for λ_ex_ = 300 nm; **—for λ_ex_ = 330 nm; ^sh^—shoulder. Values marked in bold are the values for the highest intensity emission band.

## Data Availability

The data that support the findings of the current study are listed within the article.

## Supplementary Materials

The following supporting information can be downloaded at: https://www.mdpi.com/article/10.3390/ijms232113174/s1.
