# Supplementary material for: Optical and Flame-Retardant Properties of a Series of Polyimides Containing Side Chained Bulky Phosphaphenanthrene Units
Source: Int J Mol Sci. 2022 Oct 29;23(21):13174. doi: 10.3390/ijms232113174 (PMC9656655; doi:10.3390/ijms232113174)
Supplement: Supplementary file 1 [file ijms-23-13174-s001.zip › ijms-1974984-supplementary.pdf]

SUPPORTING INFORMATION **for**

Optical and Flame-Retardant Properties of a Series of Polyimides Containing Side Chained Bulky Phosphaphenanthrene Units

Mihaela Homocianu [1], Diana Serbezeanu [1], Tăchiță Vlad-Bubulac [1]*, Mihai Brebu [1] and Gabriela Lisa [2]

[1] "Petru Poni" Institute of Macromolecular Chemistry, Aleea Gr. Ghica Voda, 41A, 700487 Iasi, Romania

[2] Department of Chemical Engineering, Faculty of Chemical Engineering and Environmental Protection, Gheorghe Asachi Technical University of Iasi, Bd. Mangeron 73, 700050 Iasi, Romania

* Correspondence: tvladb@icmpp.ro

Figure S1. Absorption spectra of **PI (1-4)** in $CHCl_3$ (a), NMP (b) and DMAc (c) solutions.
Figure S2. Emission spectra of **PI-2 (a), PI-3 (b),** and **PI-4 (c)** in DMF solution.
Figure S3. Emission spectra of **PI-1 (a), PI-2 (b), PI-3 (c), PI-4 (d),** in $CHCl_3$ solution
Figure S4. Emission spectra of **PI-1 (a), PI-2 (b),** and **PI-3 (c)** in NMP solution.
Figure S5. Emission spectra of **PI-1 (a), PI-3 (b),** and **PI-4 (c)** in DMAc solution.
Figure S6. Transmission spectra for **PI (1-4)** prepared thin films.

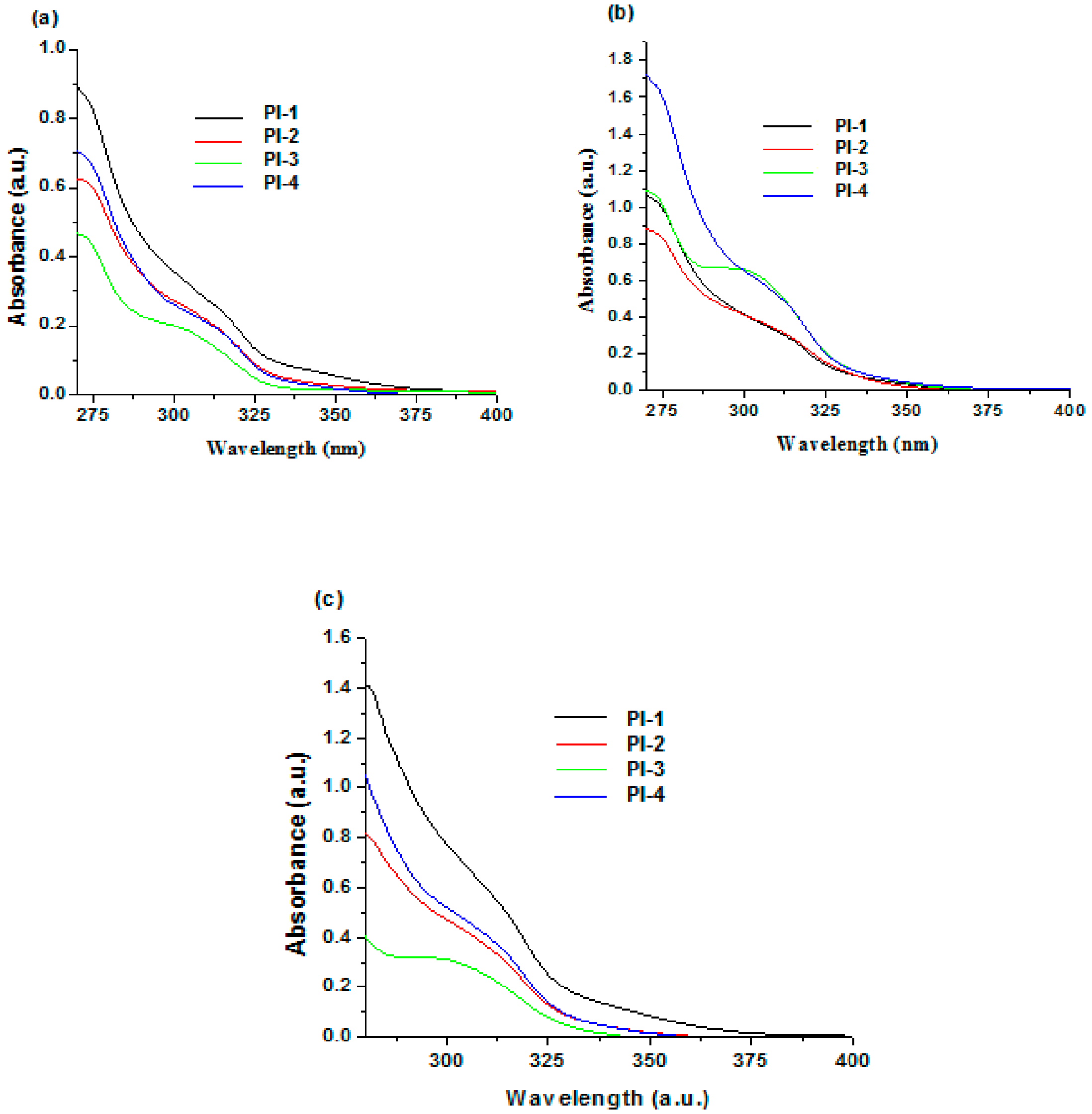

Figure S1. Absorption spectra of **PI (1-4)** in $CHCl_3$ (a), NMP (b) and DMAc (c) solutions.

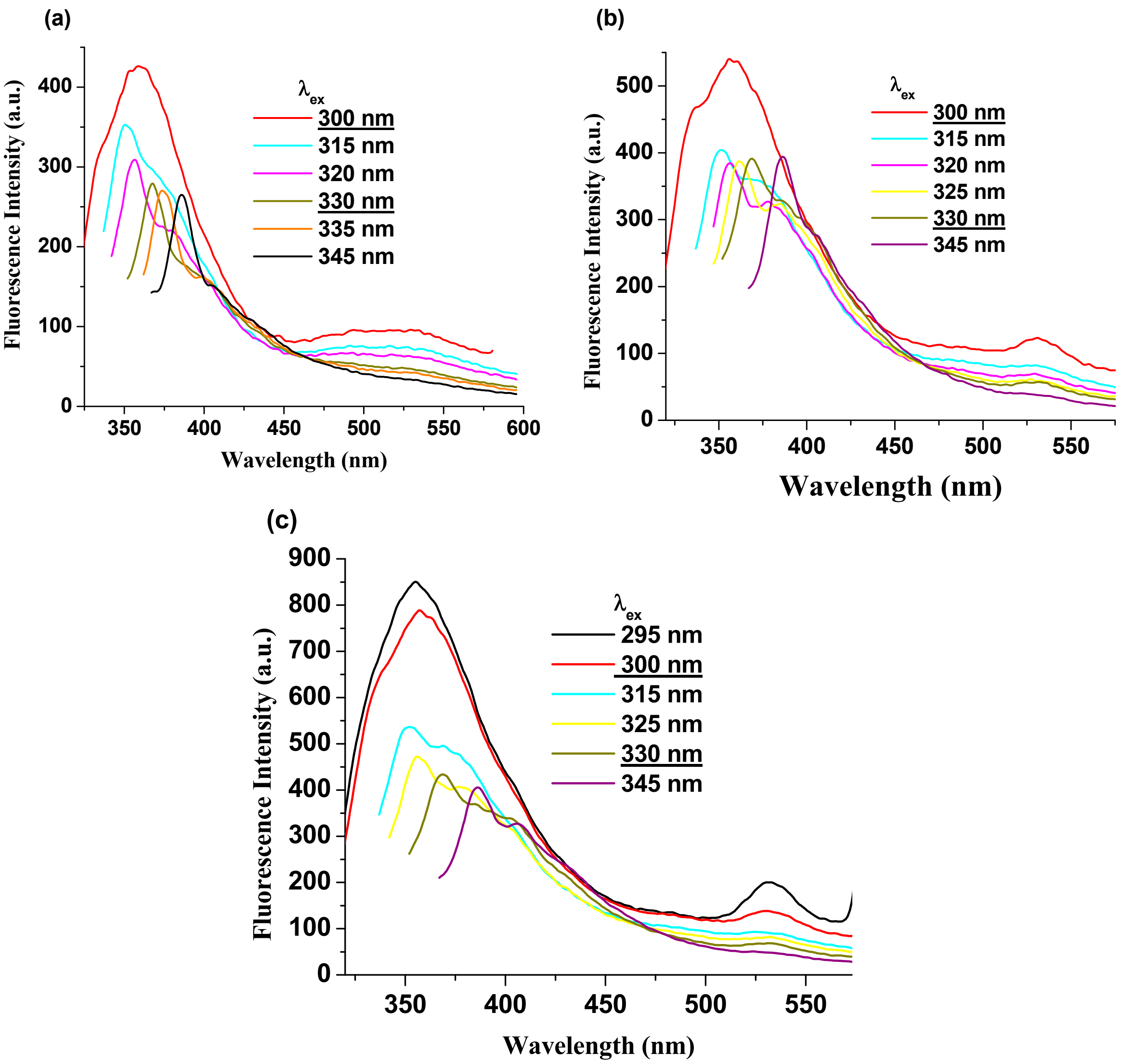

Figure S2. Emission spectra of **PI-2 (a), PI-3 (b),** and **PI-4 (c)** in DMF solution.

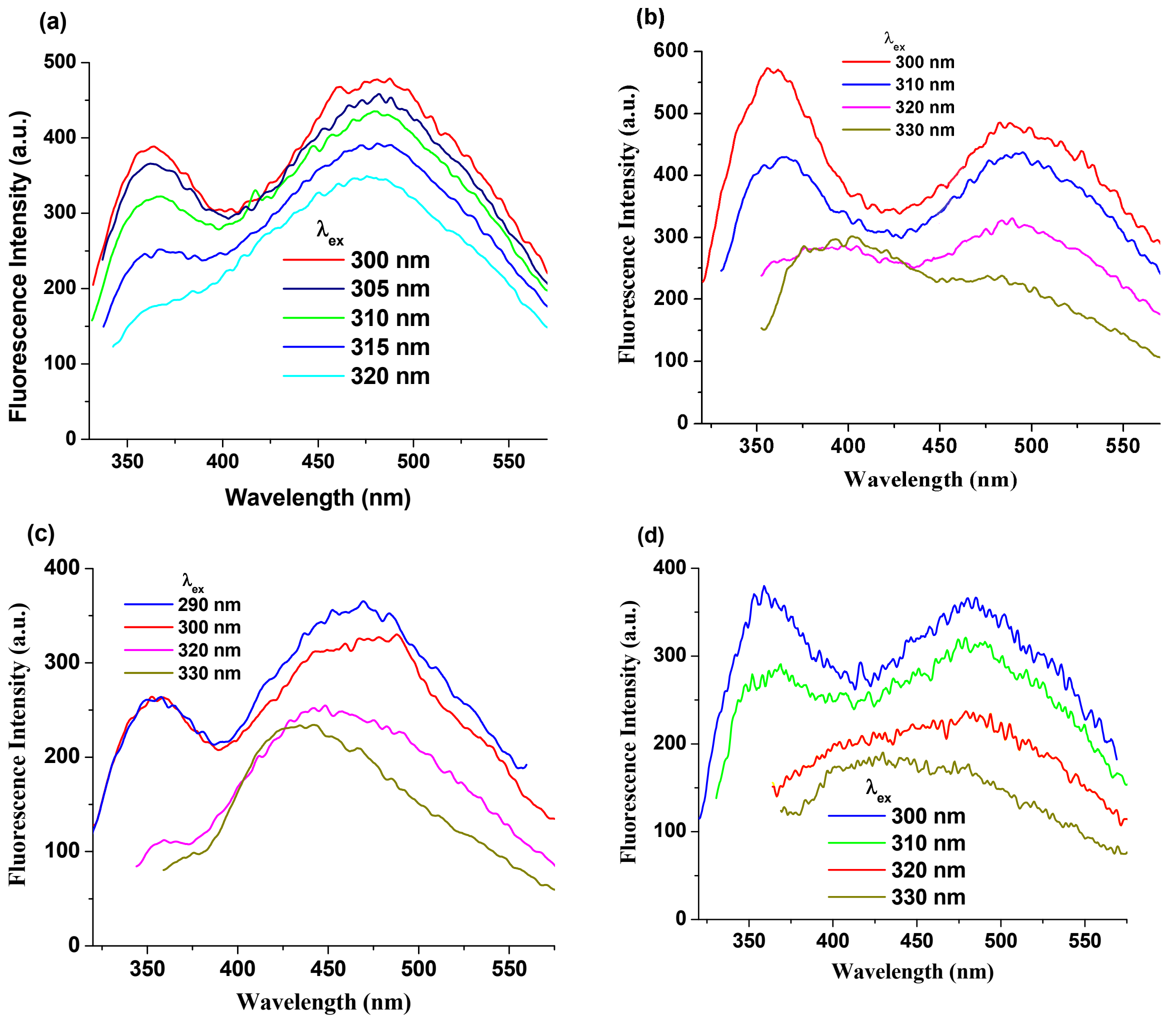

Figure S3. Emission spectra of **PI-1 (a), PI-2 (b), PI-3 (c), PI-4 (d),** in $CHCl_3$ solution

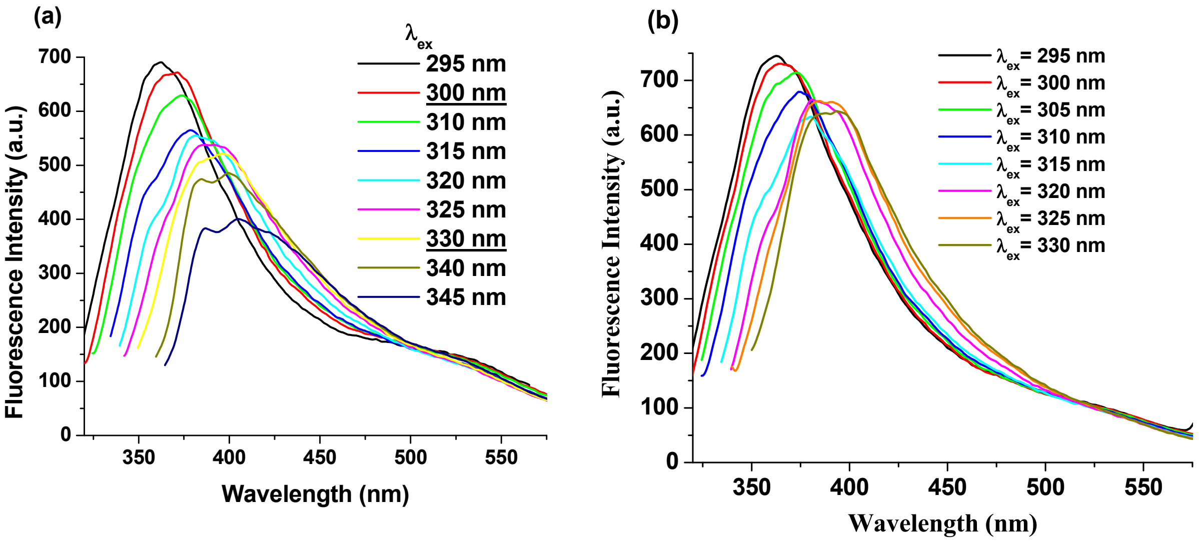

Figure S4. Emission spectra of **PI-1 (a), PI-2 (b),** and **PI-3 (c)** in NMP solution.

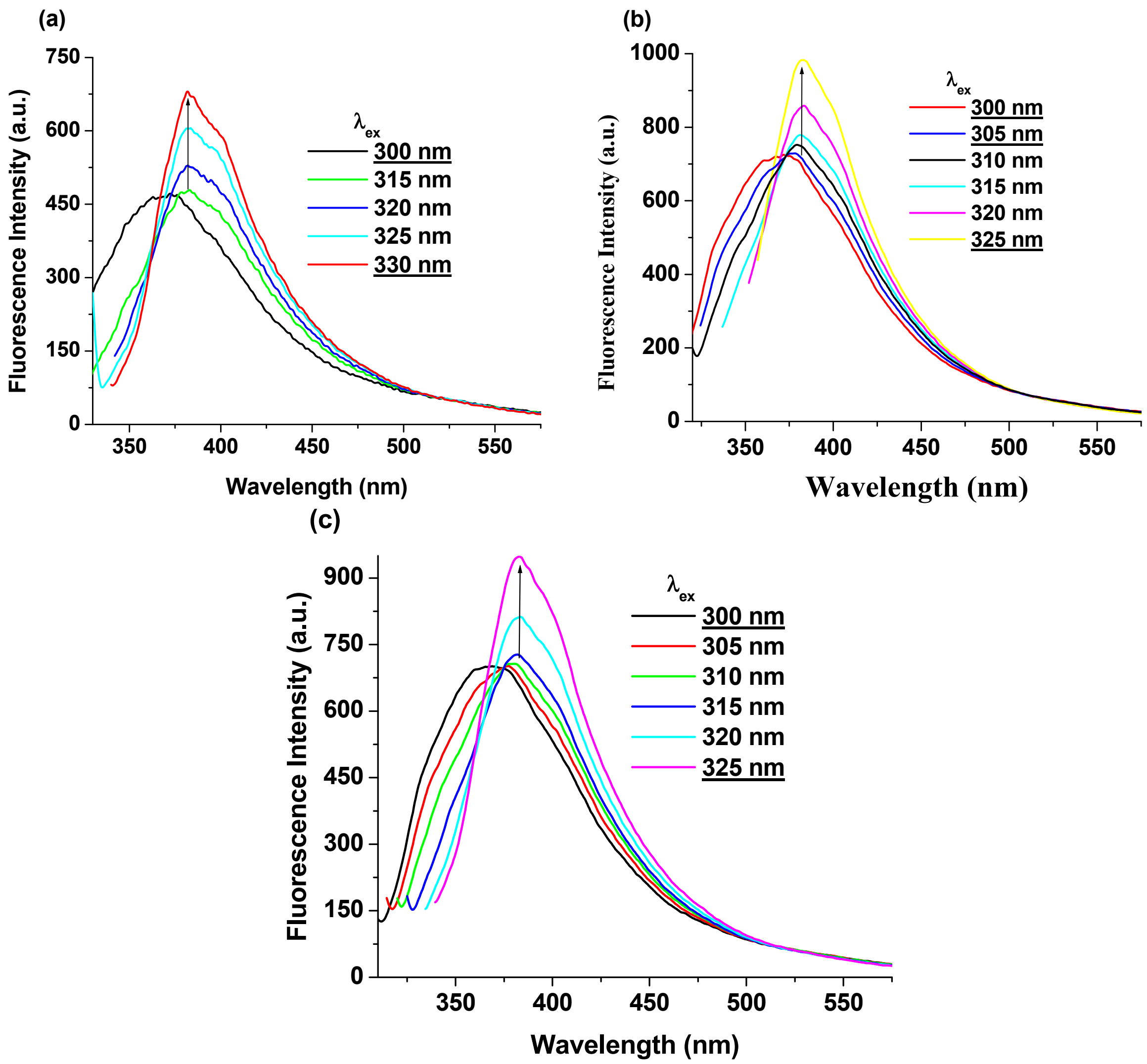

Figure S5. Emission spectra of **PI-1 (a), PI-3 (b),** and **PI-4 (c)** in DMAc solution.

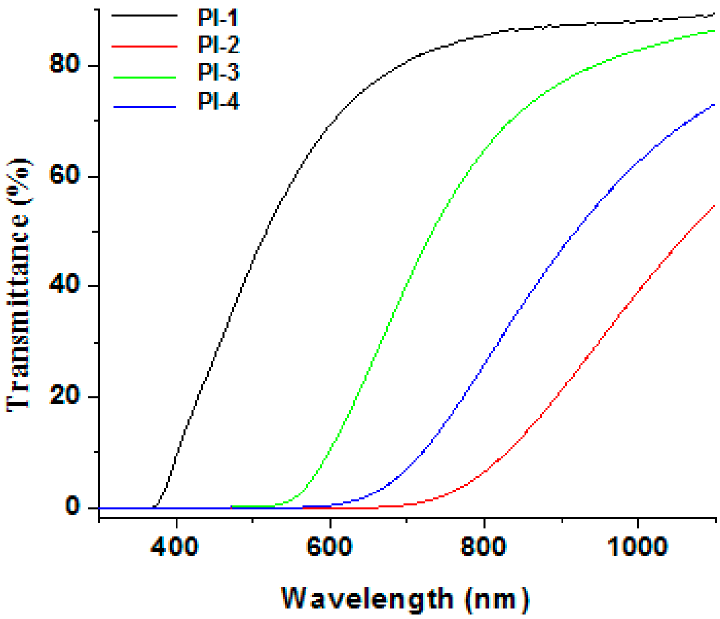

Figure S6. Transmission spectra for **PI (1-4)** prepared thin films
